# The Microscope

**Published:** 1890-02

**Authors:** F. E. Yoakum

**Affiliations:** Late of Greenville, Texas, Now of Shreveport, La.


For Daniel's Texas Medical Journal.

### THE MICROSCOPE AND SOME OF ITS USES IN DIAGNOSIS OF DISEASES.

F. E. YOAKUM, M. D., LATE OF GREENVILLE, TEXAS, NOW OF SHREVEPORT, LA.

[Read before the North Texas Medical Association, June, 1889.]

IN the arts, as well as in scientific investigations, the microscope is used for the examination and preparation of delicate work. The jeweler, the engraver, and the miner find a simple microscope essential to their employments. In commerce the microscope has been used to detect adulterations in articles of food, drugs and in all manufactured articles.

The mineralogist and geologist cannot afford to do without it. The chemist recognizes minute quantities and reactions, which could be detected only with the microscope. And in biology the wonderful power of the microscope finds its widest range. If we see not nature in her undress, we trace the elementary warp and woof of her majestic drapery—in vegetable and animal physiology we see by its means, not only the elementary unit, the foundation stone of the building, but also chambers and laboratories in the animated temple, which we should never have suspected, tissues and structures not otherwise discoverable; not to speak of species innumerable and invisible to the naked eye. In medical science and jurisprudence the contributions of microscopy have been so numerous that constant study in this department is needed by the physician who would excel, or even keep pace with the progress of his noble profession. Microscopy may well and truly be called the guiding genius of medical science. Suppose a patient should ask this intelligent association of doctors: "What is the cause of my sterility?" Could any one give him a clear answer without the aid of the microscope? I think not; you might examine the wife and find impediments to a fruitful copulation, but in the majority of cases you will find the trouble lies within the semen of the male, the living moving principle or micro-organism—called the "spermatozoa." And by this same means you can by closely examining the urine detect and diagnose masturbation, the patient's word to the contrary, notwithstanding.

And no physician, it matters not how learned he may be, will dare to tell you the abnormal constituents of urine; but with the microscope he will tell you with as much certainty as anything in the medical sciences, whether or not the pathological lesion is in the kidneys or bladder, or whether it is this great channel trying to carry off the diseased particles from other parts of the body. And let me urge you physicians, make yourselves acquainted with this powerful means of diagnosing correctly. Chronic diseases, and a thorough knowledge of urinology is the only clear way out of the mist and fog. How many catarrhal troubles of the bladder that are being treated all over the country for kidney diseases? No one can answer. And with a little study of urinology and a little time with the microscope the diagnosis is made clear—I urge this as it is woefully neglected. You can detect the smallest amount of sugar in the urine with less trouble with the microscope than by any other means. Also the excess of the triple phosphates or the urates, and renal tube casts, (which is the only pathognomonic symptom of chronic Bright's disease). I know that some of you may question this last statement, but I lay it down and leave it to you to find the falseness or truthfulness of it. How are you to diagnose for a certainty phthisis pulmonalis in the first stages? The microscope is the only means by which the intelligent physician can arrive at the correct diagnosis, and that always is closely connected with a thorough physical examination. I will now give some of the practical uses of the microscope in the examination of the secretions of the body. And I will not speak in detail of Dr. George Dock's, of Galveston, Texas, discovery or investigations in malarial fevers; his most excellent paper on this subject, read before the late Texas State Medical Association has not been published yet.* I await it with a great deal of interest, as it will be full of thought and food for the progressive physician. He claims that the "malarial micro-organism has been found in different parts of the world by several investigators of great reputation—and are found in the greatest abundance during the chill."

First Ex.—Will be the urine. We will examine the urine for epithelium tube casts, mucus, pus, blood disks, spermatozoa and the crystalizable substances.

Second Ex.—Will be milk. This is a great unexplored field for the progressive physician. There are thousands of children being torn away from the world to-day on account of our lack of knowledge in this field. Not only ought we to be able to make a microscopical examination but we should be able to make a close chemical examination of this all-important food.

Third Ex.—Saliva and Sputum. This is impossible to do without the use of the microscope.

Fourth Ex.—Of vomited matters. The most ignorant mind can see the importance of even a very low power microscope for this matter.

Fifth Ex.—Of intestinal discharges. Some of the most ludicrous mistakes have been made right on this line, where the physician did not have the microscope to aid him in arriving at conclusions. Taking for, and believing that potato peelings and

*[It is in the Transactions of 1889.—Ed.]

grapeskins, etc., are epithelium and mucous membranes. A good physician brought to my office one day "a large mucous patch," as he called it; and had given the child up to die; and when I examined the "patch" with a low power I found it to be an Irish potato peeling.

Sixth Ex.—Vaginal discharges. Is a field that, as yet has been little cultivated, but is one of especial interest to the gynecologist.

Seventh Ex.—Now we come to the broadest and most useful field for the microscope. In the etiology of diseases or the knowledge of the causes of diseases. Here the microscope will become indispensable in the numerous external causes of disease, such as physical or organic impurities in the atmosphere, soil, water and food, vegetable or animal parasites and disease germs, with their relation to epidemic or endemic disorder— all require the use of this powerful instrument. And I shall now skip to the last paper, hoping in the future to fill out between. The examination for bacteria (or "bug" as some of my good friends call them). Bacteria are extremely minute. And to get it well before your minds I will do so by comparison. A red blood corpuscle is .3500 to .4500 of an inch in diameter, and now it would take 750,000 to 1,000,000 bacteria (or "bugs") cells to equal one blood-cell or globule; and some species fall far below this size while others would excel it. The rapidity with which they multiply, small as they are, is by no means insignificant, "Cohn has that a species undergoing fission once an hour, which is not too high an estimate for some species under favorable circumstances, nearly 17,000,000 offsprings at the end of twenty-four hours, while in about a week the number could be represented only by fifty-two figures, so that is practically meaningless to the ordinary mind." And further he adds for comparison, "if they were twice the size, and reproduced this fast they would produce 928,-000,000 cubic miles, the volume of the ocean, in the short space of five days, if none were destroyed." What wonder if the germs of putrification and disease are well nigh omnipresent. Practically, how easily will these small bacteria (or "bugs") be transmitted by the physician and surgeon, and especially the deadly puerperal bacteria. Dr. Foster, of Amsterdam, and his pupil, Wassing, "tound that it was no easy matter to disinfect the hands. Thirteen men were examined in this way (with the microscope) after they had most painfully, and diligently disinfected fingers, etc., according to prescribed methods in vogue; the time employed to brush, disinfect and trim the hands varies from two to ten minutes, the solutions used here, carbolic acid and sublimate combined with soap, and only one doctor's hands found to be perfectly clean and free from bacteria. [Annals of Surgery, February, 1888, page 153.]

Gentlemen be careful about your hands and persons, and study the microscope every day of your professional lives. Who knows but what my friend Dr. Fort may find a perfect solution to "Maternal Impressions" by this wonderful instrument. It may be in an undeveloped ovum; we don't know, but scientific investigation may develop the facts in the case.

I would refer the student for further information to a paper read before the American Academy of Science by Samuel W. Nelson, M. D., of Boston, Mass., and published in the "Journal of American Medical Association," May 25, 1889. Also to a paper read before the Texas State Medical Association, in 1889, at San Antonio, Texas, by Dr. George Dock, of Galveston, Texas.

